# Characterization and antidiabetic activity of cinnamon essential oil emulsion encapsulated by complex coacervation between gelatin and soluble fraction of tragacanth gum

**DOI:** 10.1016/j.fochx.2025.103044

**Published:** 2025-09-18

**Authors:** Fateme Amani, Fatemeh Rafieian, Azra Salehi, Mohammad Sadegh Damavandi, Moein Shirzad, Hajar Akbari, Atefe Rezaei

**Affiliations:** a Department of Food Science and Technology, School of Nutrition and Food Science, Isfahan University of Medical Sciences, P.O. Box: 81746-73461, Isfahan, Iran; b Nutrition and Food Security Research Center, Isfahan University of Medical Sciences, Isfahan, Iran; c Department of Food Hygiene and Quality Control, School of Nutrition and Food Sciences, Shiraz University of Medical Sciences, Shiraz, Iran; d Department of Microbiology and Virology, Faculty of Medicine, Hasheminejad Hospital, Mashhad University of Medical Sciences, Mashhad, Iran; e Antimicrobial Resistance Research Center, Mashhad University of Medical Sciences, Mashhad, Iran; f Department of Clinical Biochemistry, Faculty of Medicine, Babol University of Medical Sciences, Babol, Iran; g School of Life Sciences, Plant Proteins and Nutrition, Technical University of Munich, Freising, 85354, Germany

**Keywords:** Cinnamon essential oil, Complex coacervation, Gelatin, Soluble fraction of tragacanth gum, Antidiabetic activity

## Abstract

Complex coacervates of gelatin (GE) and the soluble fraction of tragacanth gum (STG) at ratios of 1:2, 1:1, and 2:1 were developed to encapsulate cinnamon essential oil (CEO). These coacervates were assessed for particle yield, encapsulation efficiency, and encapsulation yield. GE2:STG1/CEO50 %, GE1:STG1/CEO25 %, and GE1:STG1/CEO50 % showed the highest encapsulation efficiency, with the latter also exhibiting the highest encapsulation yield, identifying it as the optimal formulation. FTIR confirmed intermolecular interactions, while microscopy showed rough surfaces without cracks. Thermogravimetric analysis revealed improved CEO thermal stability. Toxicity assay results showed that encapsulation reduced the cytotoxicity of CEO. CEO release was higher under simulated intestinal (42.63 %) than gastric (34.91 %) conditions. The encapsulated CEO inhibited α-amylase and α-glucosidase activities dose-dependently. These findings suggest that GE–STG coacervates provide an effective and biocompatible delivery system for CEO, offering improved stability, controlled intestinal release, and enhanced antidiabetic potential.

## Highlights

• Complex coacervates of gelatin and the soluble fraction of Tragacanth gum were prepared.
• The prepared complex was used as an encapsulant for cinnamon essential oils (CEO).
• The encapsulated CEO had *in vitro* anti-diabetic activity.

## Graphical abstract

**Figure:**
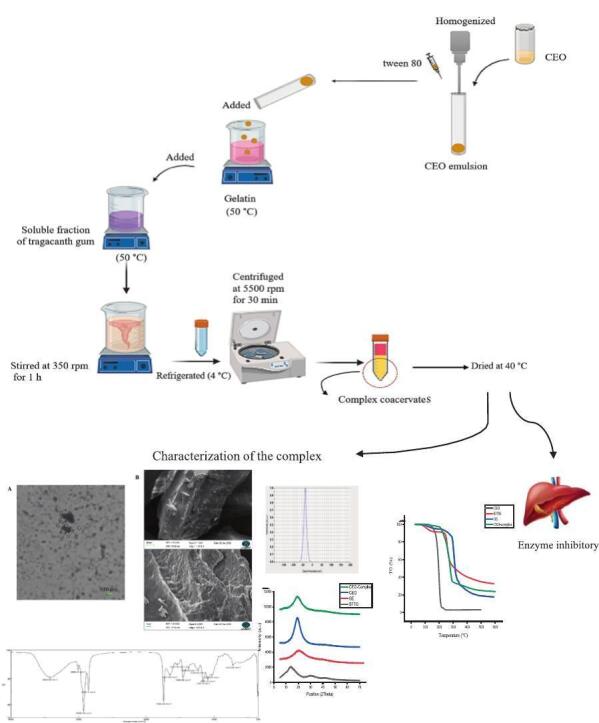

## 1 Introduction

Diabetes mellitus is a chronic and metabolic disease, caused by the body's inadequate production of insulin or the peripheral tissues' resistance to this hormone. Interests in treating diabetes mellitus by natural remedies have risen because of their lower side effects than synthetics (Ping et al., 2010). Since ancient times, the use of aromatic plants and their extracts in traditional medicine has been practiced (Edris, 2007). Cinnamon is a popular and useful flavoring agent that has been widely consumed as an herbal medicine for centuries. Nowadays, cinnamon and its derivatives are used in a wide range of commercial applications such as the food and drug industries because of its well-known anti-diabetic, anti-inflammatory, antibacterial, and antioxidant properties (Medagama, 2015; Sahib, 2016). These beneficial properties are attributed to bioactive compounds in cinnamon essential oil (CEO), such as cinnamaldehyde (El-Baroty et al., 2010; Li et al., 2013). Scientists carried out various research on the effect of the CEO on diabetes. Based on the research carried out by Mishra et al. (2010), the presence of diverse bioactive substances in CEO, especially cinnamaldehyde, can reduce the oxidant stress-related renal damage seen in alloxan-induced diabetic rats (Mishra et al., 2010). Moreover, Ping et al. (2010) found that the CEO may improve glucose balance and the immune response of pancreatic islets β-cells. Apart from these, the CEO can regulate blood glucose levels and lipids (Ping et al., 2010). EOs are unstable with very high sensitivity to external factors, such as oxygen, humidity, light, and temperature. The use of volatile essential oils in various industries has faced challenges due to their volatility, high reactivity, sensitivity to oxidation, and poor solubility in water (Mukurumbira et al., 2022). In order to address these limitations, the method of microencapsulation is frequently employed to protect the functional and biological properties and to regulate the release of these compounds. Microencapsulation involves the process of coating particles with an encapsulating agent, which serves as a protective barrier and effectively isolates the core material from the surrounding environment (Sousa et al., 2022).

Among all encapsulation strategies, complex coacervation is an emerging method, which is obtained when two biopolymers with opposite charges interact in an aqueous solution. Two heterogeneous phase are produced as a result of intermolecular interaction between polymers, and the sedimented phase is known as coacervate (Ferreira & Nicoletti, 2021). Usually, protein and carbohydrate are utilized as wall materials in complex coacervation. In the process of complex coacervation, polysaccharides' carboxyl groups interact with proteins' amino groups and form an amide containing complex (Naderi et al., 2020). Tragacanth gum)TG) is a strong acid-resistant emulsifier, used as a natural, safe food and drug additive. It is a heterogeneous, branched, and anionic polysaccharide with two major water-soluble (called tragacanthin) and water-swellable (called *tragacanthic acid* or bassorin) fractions (Karimi & Mohammadifar, 2014; Mohammadifar et al., 2006). TG is a regionally abundant hydrocolloid, particularly in Iran, which supplies about 70 % of the global demand, making it a cost-effective option compared to imported gums in Asia (Nejatian et al., 2020). The soluble fraction of TG exhibits lower interfacial tension than its water-swellable counterpart (bassorin), enabling the formation of stable and homogeneous microemulsions with smaller droplet sizes (Karimi & Mohammadifar, 2014). Compared with other common carriers such as chitosan and whey protein, TG offers distinctive structural advantages: its highly branched polysaccharide backbone provides strong emulsification capacity without the need for chemical modification, while its natural origin ensures safety and biocompatibility. Furthermore, TG has demonstrated effective controlled release properties (Shabbir et al., 2024) and high encapsulation efficiency (Navab et al., 2024; Oliveira et al., 2022), highlighting its potential as a promising novel wall material for bioactive delivery systems.

Gelatin (GE) is a biodegradable protein from collagen hydrolysis and a common stabilizer in food and pharmaceutical industries. Because of its polypeptide structure, it can bond with oppositely charged polymers and be utilized as an encapsulating agent (Bastos, Vicente, et al., 2020; Jannasari et al., 2019b; Muhoza et al., 2019). The antidiabetic activity of different essential oils encapsulated in various types of carriers was evaluated by researchers. Encapsulated CEO in whey protein resulted in elevating antioxidant activity and alleviating oxidative stress in diabetic rats (Mohammed et al., 2020). Additionally, Siahbalaei et al. (2020) reported strong antioxidant activity of four different essential oils incorporated into pectin-gelatin composite against glucose oxidation and their antidiabetic effects against amylase and glucosidase activities (Siahbalaei et al., 2020).

Therefore, the novelty of this study lies in introducing TG as a novel, cost-effective, and acid-stable wall material, and its synergistic combination with GE to form complex coacervates for CEO encapsulation. This GE–TG system not only enhances the thermal and gastric stability of CEO but also enables targeted intestinal release, which is particularly advantageous for improving its antidiabetic efficacy.

The present study was conducted with the aim of CEO encapsulation and evaluating the *in vitro* antidiabetic activity of the encapsulated CEO. Different amount of CEO was encapsulated in complex coacervates of GE and soluble fraction of tragacanth gum (STG) with various ratios. The best sample was chosen based on encapsulation efficiency (EE)/yield (EY) and was characterized by *Fourier*-transform infrared spectroscopy (FTIR), optical, and *scanning electron microscopes* (SEM), *X-ray diffraction* (XRD) analysis, zetasizer, and *thermogravimetric analysis (*TGA). The release and the antidiabetic activity of the produced complex were also investigated.

## 2 Materials and methods

### 2.1 Materials

CEO was obtained from Givaudan Company (Switzerland). TG was prepared from a market located in Isfahan, Iran. The prepared TG was previously characterized in a laboratory in the Department of Food Science and Technology, College of Agriculture & Natural Resources of Uni*v*ersity of Tehran. The range of its molecular weight was 0.18 × 10^6^–1.6 × 10^6^ g/mol, and its soluble to insoluble fraction ratio was 35/65. The amount of moisture, ash, nitrogen, and uronic acid was 12 %, 2.8 %, 0.088 %, and 17.1 %, respectively. The percentage of arabinose, xylose, glucose, fucose, galactose, rhamnose, and galacturonic acid was 1, 32, 1, 23, 1, 1, and 37 %, respectively. Type B GE (with a bloom of 225 g) was provided by Amstel Company (Netherlands). Hydrochloric acid (HCl), Tween 80 (emulsifier), n-hexane, acetic acid, and *p*-nitrophenyl-α-D-glucopyranoside (p-NPG) were supplied by Merck (Germany). Cellulose membrane dialysis tube (cut-off 12–14 kDa) was obtained from Sigma-Aldrich (Germany).

### 2.2 Preparation of STG

Firstly, TG was dissolved in distilled water to prepare a 0.25 % *w*/*v* solution. To complete hydration, the TG solution was refrigerated overnight. To separate the soluble part of TG, the solution was centrifuged (MPW-206R, MPW Med. Instruments, Poland) at 5500 rpm for 1 h. Then, supernatant was separated as soluble fraction of TG and dried at 50 °C (Amani, Rezaei, Akbari, et al., 2022).

### 2.3 Preparation of CEO emulsion

For the preparation of CEO emulsion, different amounts of CEO (25 % and 50 % based on total solid content) and tween 80 (1 % based on CEO content) were gently added to distilled water and homogenized (D-500 homogenizer, Dragon Lab, *Beijing,* China) at 10000 rpm for 5 min (Amani, Rezaei, Akbari, et al., 2022).

### 2.4 Preparation of the CEO complex

All samples were prepared based on the method described by Hernández-Nava et al. (2020) with some modification (Hernández-Nava et al., 2020). In brief, GE and STG solutions (1 % *w*/*v*) were prepared in distilled water at 50 °C, separately and then different ratios of GE: STG and CEO emulsion were formulated according to Table 1. The emulsion of the CEO was homogenized with the GE solution at 10000 rpm for 15 min. The mixture of GE and CEO was added to the STG solution, the pH of the final mixture was adjusted to 3.6 with the addition of acetic acid (10 % *v*/v) and stirred at 350 rpm for 1 h. The prepared samples were refrigerated (4 °C) to harden the wall biopolymers. Final samples were centrifuged (MPW-206R, MPW Med. Instruments, Poland) at 5500 rpm for 30 min. Supernatant was put away, and sediment phase was separated as complex coacervate and dried at 40 °C.

**Table 1:** Result of particle yield (PY), encapsulation yield (EY), and encapsulation efficiency EE) of different samples.

Sample formulation	Result				
GE:STG	CEO%	PY%	EY%	EE%	CEO loading (mg/g)
1:2	25 %	11.42 ± 0.33^a^	11.42 ± 1.33^a^	84.165 ± 7.73^a^	250.00 ± 30.00^a^
	50%	17.25 ± 1.30^b^	8.08 ± 3.50^a^	84.68 ± 4.26^a^	234.20 ± 102.97^a^
1:1	25 %	64.15 ± 0.11^c^	13.83 ± 2.24^a^	99.06 ± 0.37^b^	53.90 ± 8.73^b^
	50%	65.65 ± 2.27^c^	51.34 ± 7.14^c^	98.32 ± 0.15^b^	391.01 ± 56.03^c^
2:1	25%	73.52 ± 0.15^d^	13.60 ± 5.56^a^	84.68 ± 4.26^a^	46.25 ± 18.91^b^
	50 %	74.03 ± 0.21^d^	47.61 ± 0.80^b^	99.27 ± 0.03^b^	321.56 ± 5.48^d^

Different letters in the same column show significant differences at *p* < 0.05.

### 2.5 Characterization of the complex

#### 2.5.1 Particle yield (PY)

The PY indicates the ratio of powder gained after drying complex coacervate to the initial weight of pure materials (GE + STG + CEO). It was measured gravimetrically and calculated according to Eq. (1):

(1) $$\mathrm{PY}\%=\left(m/m_{0}\right)\times 100$$

where m and m_0_ are the final mass of the dried powder and the initial mass of the material, respectively.

#### 2.5.2 Encapsulation efficiency (EE) and encapsulation yield (EY) of complex coacervate

The gravimetric method was used to measure EE and EY of complex coacervate (Ferreira & Nicoletti, 2021). To obtain non-encapsulated CEO (surface oil- SO) in complex coacervate, 100 mg of sample was mixed with 10 mL n-hexane in a test tube and agitated for 30 s. Then, this mixture was filtered with Whatman filter paper No. 41 and placed in a pre-weighed plate. The hexane was vaporized at 30 °C under the hood. The acid digestion method was used to determine total CEO (total oil- TO) in complex coacervate powder. For this purpose, 20 mL of HCl solution (4 N) was added to 100 mg of powder and stirred at 100 rpm and 35 °C for 1 h. Then, 13 mL of n-hexane was added and stirring at the same rate was continued at 25 °C for 5 h. The mixed solution was centrifuged at 5500 rpm for 30 min, the upper hexane phase was poured into a pre-weighed plate and n-hexane was evaporated at 30 °C under the hood. The EE% and EY% were calculated according to Eqs. (2), (3):

(2) $$\mathrm{EE}\;\left(\%\right)=\left(\left(\text{TO}-\mathrm{SO}\right)/\text{TO}\right)\times 100$$

(3) $$\mathrm{EY}\;\left(\%\right)=\left(\text{TO}/\mathrm{IO}\right)\times 100$$

where IO is the initial oil.

#### 2.5.3 FTIR spectrum of the complex

To identify the functional groups of the complex coacervate and each of its components, their FTIR spectra were recorded by an FTIR spectrometer (Jasco, Japan) in the wavenumber range of 4000–400 cm^−1^ and at a resolution of 4 cm^−1^. Before analysis, the powders of the samples were *mixed with potassium bromide* and *compressed into* a *pellet* by using a hydraulic press.

#### 2.5.4 Morphological characterization

Optical microscopy images were collected using a KERN Optics OBL 137 T241 microsope equipped with an ODC 241 camera (KERN & SOHN GmbH, Balingen, Germany).

Scanning electron microscopy (SEM; *Zeiss EVO 15*, *Oberkochen*, *German*y) was used for observing the structure and morphological characteristics of complex coacervate. One drop of complex coacervate particle dispersion was applied onto aluminum foil, attached to a metal stud by double-sided conductive tape, and dried in a *desiccator* under *vacuum* at room temperature. The samples were gold-coated using the SC7620 Mini sputter coater to produce high-quality SEM images at 8 kV.

#### 2.5.5 Zeta potential measurement

The zeta potential was measured using a zeta sizer (Bruker, Germany) at room temperature and a scattering angle of 90°.

#### 2.5.6 X-ray diffraction (XRD) analysis

XRD measurements were carried out using a diffractometer (Asenware, AW-XDM300, China) with Cu K_α_ radiation *(λ* = 1.54 Å). The working conditions were as follows: continuous mode, generator voltage/current of 40 kV/30 mA, angular region of 5–60°, and scanning rate of 0.05°/min.

#### 2.5.7 Thermal property analysis

Thermal analysis was performed on the pure materials and complex coacervate by Labsys evo thermogravimetric analyzer (*Setaram* Instrumentation, Caluire, *France*) from room temperature to 600 °C under a nitrogen atmosphere and, the heating rate of 10 °C/min.

### 2.6 Toxicity assay

MTT colorimetric assay was conducted to evaluate the effects of pure and encapsulated CEO on fibroblast L929 cells, following the method outlined by Badano et al. (2019). At first, 100 μL of cell suspension in Roswell Park Memorial Institute (RPMI)-1640 medium was seeded into each well (1 × 10^4^ cells/well) in 96-well microplates. The incubation was conducted at a temperature of 37 °C within a humidified incubator containing 5 % CO2. Following a 24–h incubation period, the previous medium was replaced with 100 μL of free and encapsulated CEO with varying concentrations in each well. The cells were then further incubated for another 24, 48, and 72 h under the same conditions. Subsequently, the supernatant was removed, and 10 μL of MTT solution at a concentration of 5 mg/mL was added to each well of the plates in the dark, followed by a 2–3 h incubation at 37 °C. Then, the old media containing MTT was removed, and dimethylsulfoxide (DMSO) (100 μL) was carefully introduced into each well as a solvent for the formed formazan crystals. The absorbance of the contents of each well was read using an ELISA plate reader (Startfix-2100, Awareness, USA) at 570 nm. The negative control consisted of 1 % DMSO, whereas the positive control was represented by doxorubicin. The IC_50_, *concentration needed for 50 % inhibition of bacterial growth* were determined by plotting the percentage of cell survival against the concentration of free and encapsulated CEO. Cell viability (%) was calculated using Eq. (4):

(4) $$\%\mathrm{Cell viability}=\frac{\text{Absorbance of treated cells}\;\left(a\right)-\text{background absorbance}\;\left(b\right)\;}{\text{Absorbance of untreated cells}\;\left(c\right)-\text{background absorbance}\;\left(b\right)\;}\times 100$$

where b = blank, and c = control.

### 2.7 CEO release from complex in simulated gastric and intestinal conditions

The *in vitro* release of CEO was investigated in simulated gastric fluid (SGF, pH = 1.2) and simulated intestinal en*v*ironment (SIE, pH = 6.8). To prepare SGF, 2 g of NaCl was dissolved in 80 mL of 1 M HCl, and the *v*olume was brought up to 1 L with distilled water. For the preparation of SIE, 6.8 g of K_2_HPO_4_ was dissolved in 77 mL of NaOH solution (0.2 M) and then diluted with distilled water to a total volume of 1 L. In the first step, 20 mg of the complex coacervate powder and 3 mL of phosphate buffer were added to the dialysis tube (6 cm). A dialysis tube was put in 20 mL of SGF and incubated at ambient temperature (37 °C) under gentle agitation (100 rpm) for 2 h. Aliquots (5 mL) were removed at specific time intervals (30 min) and replaced with an equal volume of fresh SGF. In the second step, the dialysis tube was removed from SGF and placed in 20 mL of SIF. At specific time intervals (1 h), sampling was carried out for the next 6 h (Rezaei & Nasirpour, 2019). The CEO content was evaluated by UV–Vis spectrophotometer (JASCO, Japan) at 289 nm.

### 2.8 *In vitro* antidiabetic activity

#### 2.8.1 α-**Amylase inhibitory assay**

The α-Amylase inhibitory assay was conducted following the method outlined by Worthington (1993) with some modification. At first, a specific amount of CEO (40 mg) and its equivalent of encapsulated CEO were dissolved in 50 % (*v*/v) ethanol (5 mL). Then, 1 mL of this solution and 1 mL of α-amylase solution (1 U/mL) were mixed and incubated for 10 min at 25 °C. In the next step, 1 mL of 1 % *w*/*v* starch solution in 0.1 M potassium phosphate buffer (pH 6.9, containing 0.006 M NaCl) was added to the mixture. The mixture was placed in an incubator at 25 °C for 10 min and then, the reaction was terminated by adding l mL of 3, 5-dinitrosalicylic acid (DNS) colour solution. After boiling the mixture for 10 min and cooling it to the room temperature (25 ± 5 °C), the absorbance was measured at 540 nm using a spectrophotometer (*Cecil BioQuest 2501 CE*, Bath, England). Control and blank contained phosphate buffer instead of the sample (in the former) and instead of the enzyme (in the latter) were prepared. The sample was diluted in buffer to give a final concentration of 8 mg/mL, 4 mg/mL, 2 mg/mL, 1 mg/mL, and 0.5 mg/mL. The anti-diabetic activity was determined through the inhibition of α-amylase, which was expressed as the inhibition percentage and calculated by the following Eq. (5) (Worthington, 1993):

(5) $$\text{Inhibition}\;\left(\%\right)=\left(\frac{\left(\mathrm{Abs}\;\mathrm{control}-\left(\mathrm{Abs}\;\mathrm{sample}-\mathrm{Abs}\;\mathrm{blank sample}\right)\right)}{\mathrm{Abs}\;\mathrm{control}}\right)\times 100$$

#### 2.8.2 α-**Glucosidase inhibitory assay**

The α-Glucosidase inhibitory test was conducted following the method described by McCue et al. (2005) with some modifications. For this purpose, a specific amount of CEO and its equivalent of encapsulated CEO were dissolved in 50 % (*v*/v) ethanol. In the second step, 40 μL of 0.1 M potassium phosphate buffer (pH 6.9) was added to 10 μL of the prepared sample in a 96 well plate. After that, 100 μL of α-glucosidase solution at a concentration of 1 U/mL was added to each well and incubated at 25 °C for 10 min. Next, an appropriate amount (50 μL) of the *p*-nitrophenyl-α-D-glucopyranoside (p-NPG) solution, which was prepared by mixing 2 mM p-NPG in 50 mM sodium phosphate buffer (pH 6.9), was added to the mixture, and the 1st absorbance (0 min) was read at a wavelength of 405 nm. The 2nd absorbance was measured at the same wavelength but after 5 min incubation at 25 °C. A control containing 50 μl of phosphate buffer in place of the sample was prepared. The α-glucosidase inhibitory activity was determined using Eq. (6) (McCue et al., 2005):

(6) $$\text{Inhibition}\left(\%\right)=\frac{\left(\mathrm{Abs}\;\mathrm{control}\;5\min-\mathrm{Abs}\;\mathrm{control}\;0\;\min\right)-\left(\;\mathrm{Abs}\;\mathrm{sample}\;5\min-\mathrm{Abs}\;\mathrm{sample}\;0\min\right)}{\left(\mathrm{Abs}\;\mathrm{control}\;5\min-\mathrm{Abs}\;\mathrm{control}\;0\;\min\right)}100$$

#### 2.8.3 Glucose uptake assay

Glucose uptake assay was done similar to Gupta et al., with some modifications (Gupta et al., 2009). For this purpose, monolayer of 3 T3-L1 was grown in growth media composed of Dulbecco's Modified Eagle Medium (DMEM) with glucose, fetal bovine serum, and two antibiotics, penicillin, and streptomycin, at concentrations of 4.5 g/L, 10 %, 100 IU/mL, and 100 μg/mL, respectively. After that, cells were incubated at 37 °C in a humidified incubator with ambient oxygen and 5 % CO_2_. For the glucose uptake assay, cells were cultured on 6 well plates and incubated at 37 °C for 24 h in a CO_2_ incubator. After semi-confluent monolayer formation, the culture was renewed with serum-free DMEM containing bovine serum albumin (BSA, 0.2 %) and then placed in a CO_2_ incubator at 37 °C for 18 h. Then, the media was removed, cells were rinsed once with Krebs-Ringer phosphate)KRP(buffer and treated with standard drug (metformin, 25 μg/mL), complex (125 μg/mL), insulin (10 U/mL), and insulin (10 U/mL) + complex (125 μg/mL). After that, 100 μl of glucose solution (1 M) was added and incubated at 37 °C for 30 min. The supernatant was gathered, and the cells were washed three times with 1 mL ice-cold KRP buffer to stop glucose uptake. Afterward, cells were lysed by 3 consecutive cycles of freezing and thawing, and their lysates were collected for glucose assessment.

Glucose uptake was determined by subtracting the final glucose content from the initial amount in the incubated medium by the glucose oxidase-peroxidase coupled method (GOD-POD method) described by Gupta et al. (2009). For this purpose, 10 μl of the sample and 200 μL of GOD-POD reagent were mixed, and after incubation at 37 °C for 10 min, the absorbance was recorded at 550 nm wavelength. Glucose uptake (%) over control was obtained according to the following equation where control is the solution having all reagents except the test sample:

(7) $$\;\mathrm{Glucose concentration}\;\left(\mathrm{mg}/\mathrm{dl}\right)=\frac{A_{\mathrm{sample}}}{A_{\mathrm{standard}}}\times 100\;$$

### 2.9 Statistical analysis

Statistical analyses were performed utilizing the SPSS program (version 20.0; SPSS Inc., Chicago, IL, USA). The data were assessed through Analysis of variance, followed by Duncan's multiple range tests. All the results were presented as mean ± SD, and differences were considered significant at *P* values of less than 0.05.

## 3 Results and discussion

### 3.1 Particle yield, encapsulation efficiency, and encapsulation yield of complex coacervate

The values of PY, EE, and EY are collected in Table 1. As can be seen, PY% ranged from 11.42 ± 0.33 % (for GE1:STG2/CEO25 %) to 74.03 ± 0.21 % (for GE2:STG1/CEO50%). Biopolymers ratio as well as the charge balance in the complexes could be influential factors on the result of PY% (Amani, Rezaei, Damavandi, et al., 2022; Eratte et al., 2014).

High EE of CEO (> 84 %) was achieved using complex coacervation of GE and STG. Samples GE2:STG1/CEO50%, GE1:STG1/CEO25 %, and GE1:STG1/CEO50% had the highest efficiency with no significant difference between their values, but the last sample was chosen as optimal because the highest EE (51.34 %) also belonged to this sample.

The highest EE (99.27 %) was obtained for the sample GE2:STG1/CEO50%, which was higher than the EE value (91.74) reported in the study conducted by Yang et al. (2021) with chitosan/whey protein isolate as the wall materials (Yang et al., 2021).

EY reflects the total amount of essential oil retained in the final microcapsules, while EE measures the proportion of core material successfully entrapped within the wall matrix. In encapsulation processes, EE values are often higher than EY values due to the volatile nature of essential oils and losses that occur during drying (Napiórkowska et al., 2023). According to Table 1, the lower EY (51.34 %) compared with EE (98.32 %) may be attributed to several factors, including oil deposited on the surface of microcapsules during coacervate formation, which could not be fully removed by n-hexane washing (Amani, Rezaei, Damavandi, et al., 2022; Hernández-Nava et al., 2020; Jannasari et al., 2019b). Additionally, core-to-shell ratio, polymer concentration, and the coacervation procedure itself may influence the total yield (Barra et al., 2019; Jannasari et al., 2019a; Timilsena et al., 2019). Thus, while EE highlights the effectiveness of oil entrapment within the capsules, EY reflects the overall process efficiency, which can be reduced by surface oil and processing losses.

In addition, the actual loading of CEO in the microcapsules was calculated as mg of CEO per g of dried powder using the relation:

$$\mathrm{CEO}\;\text{loading}\;\left(\mathrm{mg}/g\right)=\frac{\mathrm{EY}\;\left(\%\right)\times \mathrm{CEO}\;\text{initial}\;\left(\mathrm{mg}\right)}{\mathrm{PY}\;\left(\%\right)}$$

Depending on formulation, CEO loading ranged from **46 to 391 mg/g**, with the highest value observed for **GE1:STG1/CEO50 % (∼391 mg/g).** These results are consistent with the higher EY and PY obtained for this formulation, confirming its efficiency as the optimal carrier system.

### 3.2 FTIR analysis

**Fig. 1:**
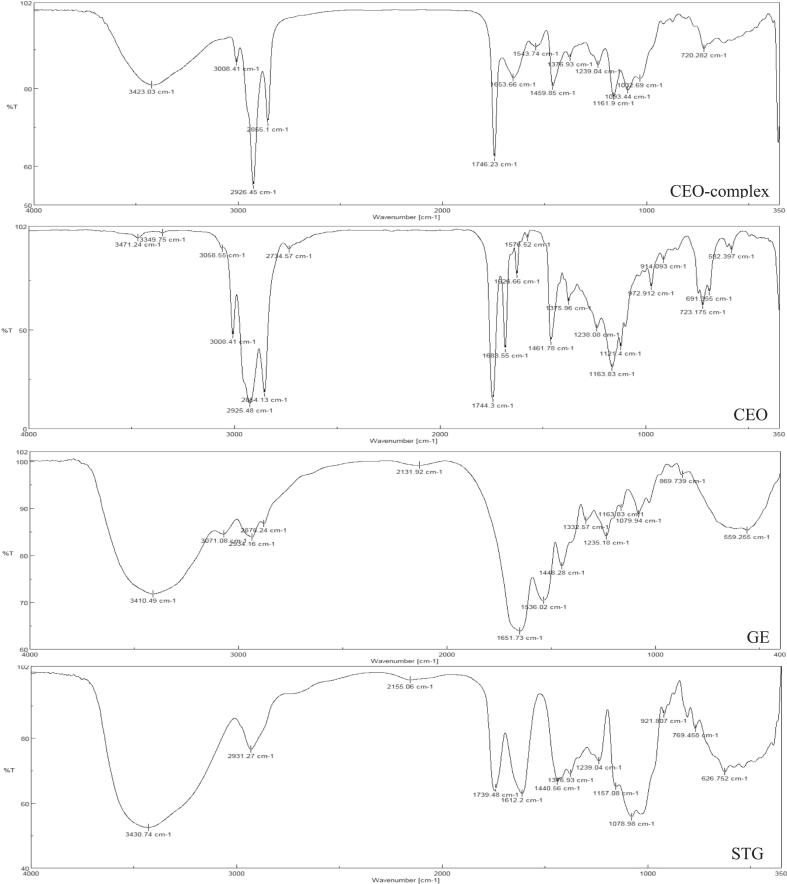
Fourier Transform Infrared (FTIR) spectra of GE, STG, CEO, and complex coacervate.

Fig.1 indicates the FTIR spectra of pure materials and CEO complex coacervates.

FTIR spectra of GE exhibited several characteristic absorption bands. The peak at 1078 cm^−1^ corresponded to C—O stretching, while another at 1163 cm^−1^ indicated C—O stretching of carboxylic acids (Devi et al., 2012) The absorption at 1235 cm^−1^ was assigned to CH₂ of glycine and amide III, representing C—N and N—H in-the plane bending of amide bonds (Theerawitayaart et al., 2019). Bands at 1430 and 1444 cm^−1^ reflected the symmetrical stretching of –COOH groups and CH₂ bending, respectively (Peng et al., 2020; Theerawitayaart et al., 2019). The amide II band appeared at 1536 cm^−1^ (N—H bending coupled with C—N stretching), while the amide I band at 1651 cm^−1^ represented the secondary structure of the protein (Devi et al., 2012; Wang et al., 2024). Amide *B* was observed at 3071 cm^−1^ (Derkach et al., 2019; Devi & Maji, 2010; Nuryanto et al., 2018; Riaz et al., 2018). In addition, peaks at 2934 cm^−1^ (C—H stretching of alkenes), 2878–2936 cm^−1^ (CH stretching vibration of proteins), and 3410 cm^−1^ (amide A and O—H stretching of water) were also evident (Hu & Yuan, 2018; Ponez et al., 2012).

For STG, the spectrum displayed a broad band at 3430 cm^−1^ corresponding to O—H stretching vibrations, along with an asymmetric methylene stretching at 2931 cm^−1^. The band at 2155 cm^−1^ indicated the presence of different carbonyl species, while 1739 cm^−1^ reflected C=O stretching of aldehydes, ketones, and carboxylic acids. Peaks at 1612 cm^−1^ (asymmetrical stretching of carboxylate groups) and at 1440–1376 cm^−1^ (symmetrical stretching of carboxylates) further confirmed the polysaccharide structure. Additional bands were observed at 1239 and 1157 cm^−1^ (C—O stretching of polyols), 1078 cm^−1^ (C—O stretching of ether groups), and 626 cm^−1^ corresponding to the pyranose ring (Asghari-Varzaneh et al., 2024; Ranjbar-Mohammadi et al., 2013).

The FTIR spectrum of CEO was characterized by distinctive signals. The absorption at 2925 cm^−1^ corresponded to C—H stretching of alkanes, while the strong peaks at 1683 and 1626 cm^−1^ indicated aldehyde carbonyl stretching, confirming the presence of cinnamaldehyde as a major component (Azadi et al., 2023). Additional characteristic bands were observed at 1575 cm^−1^ (aromatic C=C) (Alizadeh Behbahani et al., 2020), 1461 cm^−1^ (alcohol C—OH bending) (Azadi et al., 2023), and 1237 cm^−1^ (C—O—C bond of aromatic acid esters and C—OH of phenolic compounds) (Alizadeh Behbahani et al., 2020). Peaks at 973 cm^−1^ (C—H bond), 723 cm^−1^ (benzene ring C—H vibration), and 691 cm^−1^ (alkene vibration) further supported the presence of phenolic and aromatic compounds in CEO (Azadi et al., 2023).

Hydrogen bonding can form between polymer chains. The most common hydrogen bonding sites are alcohol, amide, amine, and carboxylic acid groups. Shifts in the vibrational frequencies of the functional groups, the appearance of new bands, and the changes in the intensity of the characteristic peaks in the FTIR spectra of complex coacervates suggest alterations in the chemical structures of the constituents due to *the hydrogen bonding and other* interactions between them.

Shifts in peak positions of amide A, amide I, and II from 3410, 1651, and 1536 cm^−1^ in the GE spectrum to 3423, 1653, and 1543 cm^−1^ in complex spectrum, respectively, as well as shifts in the positions of peaks associated with stretching vibrations of O—H groups and stretching vibrations of carbonyl groups from 3430 and 1739 cm^−1^ in STG spectrum to 3423 and 1746 cm^−1^ in complex spectrum, respectively, indicate their involvement in hydrogen bonding.

The appearance of CEO fingerprint regions (at 3008 and 1375 cm^−1^) or the displacement of some of them (from 2925 to 2926 cm^−1^, 2854 to 2855 cm^−1^, 1163 to 1161 cm^−1^, 1238 to 1239 cm^−1^, 1461 to 1459 cm^−1^ and 723 to 720 cm^−1^) in the FTIR spectrum of the complex can indicate the successful microencapsulation of essential oil.

### 3.3 Morphology

**Fig. 2:**
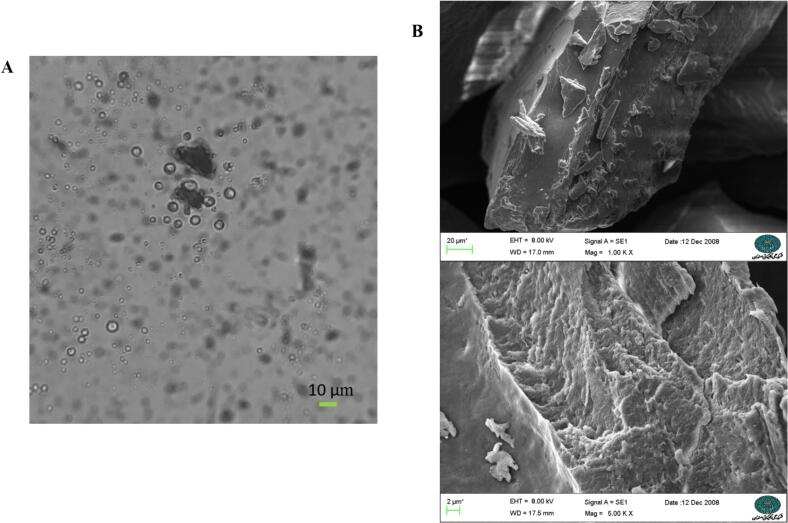
Optical microscopy image of CEO-complex (A), SEM image of CEO-complex (B).

Figs. 2A and 2B show the images captured by optical microscopy and SEM, respectively. According to Fig. 2A, the CEO droplets were trapped by the wall biopolymers. In the optical microscopy images, the microcapsules appeared spherical and intact with various diameters. In addition, the absence of agglomeration was evident. Similar findings have been reported in previous research on the encapsulation of EOs using complex coacervation (Bastos, de Sá Costa, et al., 2020; Manaf et al., 2018; Yuan et al., 2018). Chaib et al. (2021) emphasized the influential role of operating conditions such as concentrations of EO and wall materials, the nature and concentration of the cross-linking agent, and type of agitation on capsule formation in terms of number, shape, and size (Chaib et al., 2021).

The SEM image of the microcapsules (Fig. 2B) showed a rough surface with some pores but no cracks or fractures, which is a useful characteristic for oil protection. Therefore, it can be concluded that the combination of GE and STG provides a suitable wall system to protect bioactive compounds and subsequently to maintain their biological activity for a longer period of time.

### 3.4 Zeta potential measurement

**Fig. 3:**
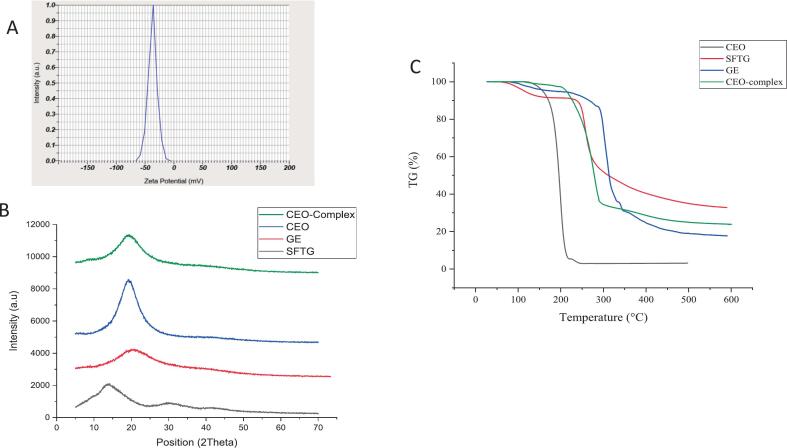
Zeta potential curve *(A),* X-ray diffraction (XRD) patterns of GE, STG, CEO, and CEO-complex (B), Thermogravimetric analysis (TGA) of GE,STG, CEO, and CEO-complex (C).

Zeta potential is a measure of the surface charge of colloidal particles, such as quacervates, in a dispersion. The high negative zeta potential (Fig.3A) (−37 mV in this case) indicates that the particles have a large negative charge on their surface, which in turn creates a strong electrostatic repulsion between the particles prevents their aggregation, and instability of the coacervate system (Amani, Rezaei, Damavandi, et al., 2022; da Silva Soares et al., 2019).

### 3.5 XRD analysis

Crystalline structures of raw materials, as well as complex coacervate, were investigated by X-ray diffraction. Sharp, *high-*intensity *peaks* in an *X-ray diffraction* (*XRD)* pattern usually indicate the presence of well-organized *crystalline* material (Abbas et al., 2023), while low crystallinity is characterized by a wider peak (Zhou et al., 2021). In the XRD pattern of pure GE (Fig.3B), a broad peak was identified at 2θ = 19.9°, assigned to amorphous structure of GE (Yao et al., 2019). *The results* described in *the* present study *are consistent with several earlier* investigations (Aguilar-Mendez et al., 2010; Radev et al., 2009). The prisitin TG showed broad diffraction peaks at 2θ values of approximately 14°, 30° and 42° (with very low intensity), which indicate that the nature of this polysaccharide is almost amorphous (Azadi et al., 2023).

In the case of the CEO, there is one broad peak at 2θ = 19.35°. CEO-complex presented an almost wide peak at around 2θ = 19.45°.

In complex coacervates, the core and wall materials can interact through various mechanisms, including electrostatic/van der Waals forces and hydrogen bonding. These interactions can cause the core and wall materials to align or aggregate in specific ways, leading to changes in the material's crystalline structure and phase composition.

### 3.6 Thermal property analysis

The TGA curves of GE, STG, CEO, and CEO-complex are shown in Fig. 3C. The weight loss (WL) of GE powder was analyzed across three temperature ranges: 66–148 °C, 284–333 °C, 333–347 °C. In the initial step, a decrease of 4.35 % in the weight of the sample was recorded, indicating the evaporation of adsorbed and bound water. Increasing the temperature from 284 °C to 333 °C led to approximately 51 % increase in WL. At higher temperatures up to 347 °C, the WL reached 69 %. The reductions of remaining mass during the last two stages are attributed to degradation by endothermic reactions of hydrolysis and oxidation. The percentage of residue at a final temperature (590 °C) was around 17.75 % (Nady & Kandil, 2018).

The thermogram of STG revealed a three-stage thermal degradation process. The TGA profile indicated an initial WL of approximately 6.9 % at 51–185 °C, which was likely due to the release of adsorbed water. This was followed by the 2nd step of WL of about 31.7 % at 209–298 °C, attributed to the degradation of the heterogeneous and highly branched structure of the two STG fractions (related to the breakdown of the main chain of STG as well as the loss of side groups including acid and ester groups). The final step, involving the complete oxidation of the polysaccharide, occurred at 324–388 °C, leaving a final solid residue of 5.3 % (Martín-Alfonso et al., 2019; Versaci et al., 2023).

The degradation of the CEO happened in two stages, the first one was in the temperature range of 121–225 °C and the second step started at around 225 °C and continued until completion at 252 °C (Amani, Azadi, Rezaei, et al., 2022).

The main decompostion of CEO-complex started at 160 °C, which continued to almost 500 °C. Finally, over 20 % of the CEO-complex did not degrade at high temperatures (632 °C). This shows that GE and SFTG complex coacervates can be a suitable wall substance for sensitive bioactive compounds such as CEO.

Table 2 displays various data obtained from the TGA and DTG curves: onset decomposition temperature (T_0_), the temperature corresponding to 10 % WL (T_10_), the temperature corresponding to 50 % WL (T_50_), the temperature of maximum decomposition rate, and the corresponding WL percentage (T_max_ and d_max_, respectively) and the mass percentage of the remaining solids at the final temperature of 600 °C which is identified by the symbole MT_600_.

**Table 2:** Parameters extracted from TGA and DTG for GE, STG, CEO, and CEO-complex.

	T_0_ (°C)	dT_10_ (°C)	dT_50_ (°C)	T_max_ (°C)	d_max_ (%)	MT_600_ (%)
GE	286.3	266.2	313.2	299.9	0.13911925	17.75
STG	73.3	236.4	311.6	256.6	0.07884	32.85
CEO	180.2	171.1	195.7	198.0885	0.22308	3.23
CEO-complex	243.5	227.3	278.4	278.3	0.0733	23.91

According to most of the parameters displayed in Table 2, the thermal stability of CEO increased after encapsulation. This occurrence can be attributed to the hydrophobic–hydrophilic interactions between the wall materials (GE and STG) and the core (CEO). Similar results were observed in a study conducted by Barzegar et al. (2016). According to their report, the encapsulation of thyme essential oil (TEO) led to its decomposition at a higher temperature than its free counterpart, reflecting its improved thermal stability (Barzegar et al., 2016).

### 3.7 Toxicity test

**Fig. 4:**
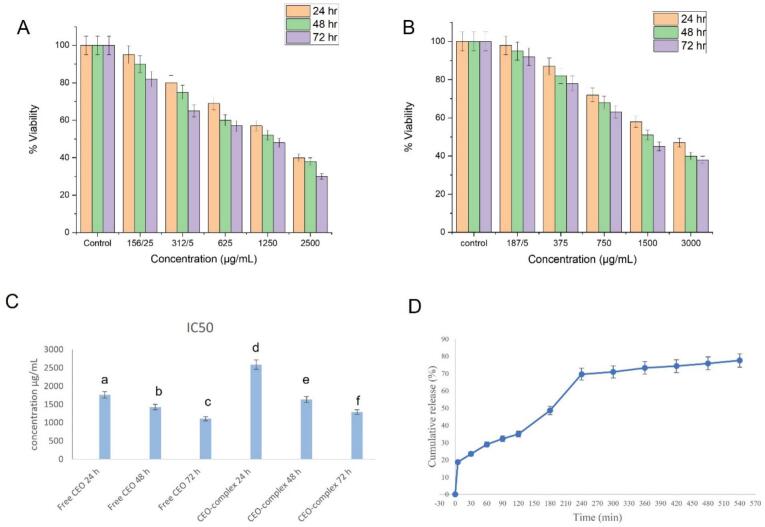
MTT assay results showing cell viability under different concentrations of free CEO (A) and CEO-complex over 72 h (B), Comparison of mean IC50 of Free CEO and CEO complex at different times. Different letters show that the *p*-value is 0.05 or lower and the results are statistically significant (C), Cumulative release of CEO in simulated gastric fluid (SGF) and simulated intestinal environment (SIE) (D).

The survival of fibroblast cells is inversely dependent on exposure time and concentration, and shows a decreasing trend with the increase of these two variables. The essential oil complex showed more cell viability in higher concentrations, which indicates a decrease in the cytotoxicity of the essential oil due to the microencapsulation process (Fig. 4A and B).

The mean IC50 of the CEO complex was higher than free CEO, and there was a significant difference between different exposure times of CEO complex and the free CEO (Fig. 4C). It was also observed in other studies that encapsulation or complexation could decrease the toxicity of the incorporated compounds such as propolis (Salehi et al., 2023), rosemary essential oil (Amani, Rezaei, Damavandi, et al., 2022) and eugenol (Nagaraju et al., 2021).

### 3.8 CEO release from the complex in SGF and SIF

The cumulative release of encapsulated CEO was surveyed in gastrointestinal conditions, and the results are shown in Fig.4D. Regarding the results, almost over 18.7 % of the CEO was released after 5 min of immersion in the SGF. This rapid *initial release* or *burst release is attributed* to the portion of the CEO that is *adsorbed* or loosely bound to the large *surface area* of the polymer. Then, the release rate decreased and was only 16.21 % in the next 115 min of exposure to gastric conditions.

At 2 h of intestinal digestion, EO release increased as a function of time, and reached to about 70 % during 2 h of immersion in simulated intestinal fluid (SIF) and to 77 % after 5 h.

The GE B protein possesses the isoelectric point (pI) at *4.8* (Milano et al., 2023). At pH < pI (in SGF), GE has net positive charges, which enable it to interact and form complex coacervates with polyanions, such as STG (Nguyen Le et al., 2021), but at pH > pI (in SIF), the amount of positively charged groups in the protein is very limited (Santos et al., 2021), and both biopolymers possess a net negative charge, which creates an electrostatic repulsion between protein–polysaccharide chains in a mixed system (Dong et al., 2023), leading to greater release of entrapped substances.

Concerning previous studies, the release results of bioactive substances from complex coacervates were approximately similar to our achieved outcomes. In another study, the release rate of black pepper essential oil from lactoferrin‑sodium alginate during exposure to simulated gastric conditions (2 h) was approximately 24.30 %, while the release of CEO was accelerated in the intestinal phase and reached 84.87 % after 2 h. It reached 84.8 % (Bastos, Dos Santos, et al., 2020).

Bioactive compounds in oil form, such as CEO, often exhibit poor absorption and limited bioavailability in the human body (Xu et al., 2023). Encapsulation not only enhances their stability under gastrointestinal conditions but also enables controlled release, thereby improving therapeutic functionality. In this study, the release rate of the CEO was higher in the intestine (42.63 %) compared to the stomach (34.91 %), which is advantageous for diabetes management. Since glucose absorption and regulation primarily occur in the small intestine, a higher release of the CEO in this region could improve its bioactivity and facilitate a more consistent hypoglycemic effect. Moreover, encapsulation protects CEO from degradation in the acidic gastric environment, ensuring greater delivery to the intestine and potentially enhancing its efficacy in controlling postprandial blood glucose levels.

As regards our results, GE and STG complex coacervates can be employed as shells to preserve core material effectively in gastrointestinal conditions for controlled release purposes.

### 3.9 *In vitro* antidiabetic assay

#### 3.9.1 α-Amylase and α*-glucosidae* inhibitory assay

α-amylases catalyze the breakdown of alpha-glycosidic bonds in glycogen and starch. Inhibition of amylase is considered an effective approach for treating carbohydrate metabolism disorders and related diseases such as tooth decay, obesity, and diabetes (Siahbalaei et al., 2020).

Glucosidase is another important enzyme that catalyzes the hydrolysis of carbohydrates like starch and disaccharides during the last stage of digestive process. Therefore, its inhibitors can delay the release and absorption of glucose, suppressing postprandial hyperglycemia, and thus play a key role in the treatment of diabetes (Dege et al., 2021; Siahbalaei et al., 2020).

**Fig. 5:**
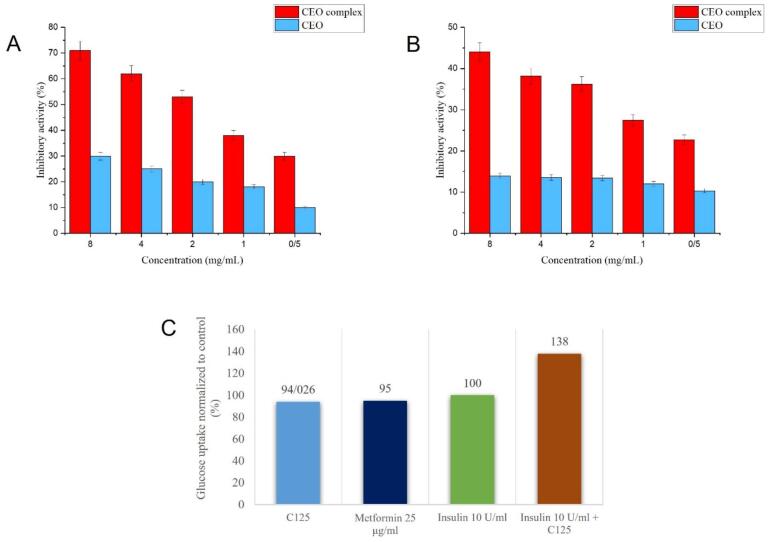
α- Percentage inhibition of α-amylase (A) and α-glucosidase (B) enzyme activity at different concentrations of free CEO and CEO-complex, Glucose uptake in 3 T3-L1 adipose cells (C).

According to Fig. 5A and 5B, encapsulating the CEO led to a significant increase in its inhibitory properties against both investigated enzymes. The effect of the encapsulated CEO on α-*amylase* and α*-glucosidae activity* was *dose-dependent from 0.5 to 8 mg/mL concentration,* in which the *inhibition* was *elevated* with *increasing concentration* of this inhibitor, and the minimum enzyme activity (71 % and 44.09 %, respectively) was obtained at 8 mg/mL.

#### 3.9.2 Glucose uptake

Fig. 5C illustrates the impact of the CEO on glucose uptake. The ability of the CEO-complex to stimulate uptake of glucose in 3 T3-L1 cells was explored by setting insulin and metformin as positive controls. In non-treated group, low glucose uptake was found. Addition of metformin, insulin, and insulin + C125 to the KRB buffer caused an increase in the glucose uptake percentage. The latter showed the best performance and the foremost restorative effect on glucose consumption (138 %).

## 4 Conclusion

In conclusion, this study demonstrated that GE–STG complex coacervates can serve as a promising wall material for CEO encapsulation. The optimized formulation (GE1:STG1/CEO50 %) significantly enhanced CEO stability, reduced cytotoxicity, and enabled targeted intestinal release, which translated into improved enzyme inhibition and antidiabetic potential. These results highlight the potential of GE–STG coacervates as a cost-effective and safe encapsulation system for bioactive compounds, with promising applications in functional foods and natural diabetes management strategies**.**

## CRediT authorship contribution statement

**Fateme Amani:** Writing – original draft, Project administration, Investigation, Formal analysis, Data curation, Conceptualization. **Fatemeh Rafieian:** Writing – review & editing, Visualization, Validation, Conceptualization. **Azra Salehi:** Writing – original draft, Investigation, Conceptualization, Formal analysis, Writing – review & editing, Data curation. **Mohammad Sadegh Damavandi:** Methodology, Investigation, Conceptualization. **Moein Shirzad:** Project administration, Methodology, Conceptualization. **Hajar Akbari:** Methodology, Investigation, Conceptualization. **Atefe Rezaei:** Writing – review & editing, Writing – original draft, Visualization, Validation, Supervision, Software, Resources, Project administration, Methodology, Investigation, Funding acquisition, Formal analysis, Data curation, Conceptualization.

## Declaration of competing interest

The authors declare that they have no known competing financial interests or personal relationships that could have appeared to influence the work reported in this paper.

## Data Availability

Data will be made available on request.
